# Documenting the absence of bovine brucellosis in dairy cattle herds in the southern region of Malawi and the associated knowledge, attitudes and practices of farmers

**DOI:** 10.4102/jsava.v92i0.2130

**Published:** 2021-08-03

**Authors:** John P. Kothowa, Ruth L. Mfune, Jacques Godfroid, Bernard M. Hang’Ombe, Martin Simuunza, John B. Muma

**Affiliations:** 1 Department of Disease Control, School of Veterinary Medicine, University of Zambia, Lusaka, Zambia; 2 Department of Animal Health and Livestock Development, Ministry of Agriculture and Food Security, Malawi Government, Lilongwe, Malawi; 3 Public Health Department, Michael Chilufya Sata School of Medicine, The Copperbelt University, Ndola, Zambia; 4 Department of Artic and Marine Biology, Faculty of Biosciences, Fisheries and Economics, UiT The Arctic University of Norway, Tromsø, Norway; 5 Department of Paraclinical, School of Veterinary Medicine, University of Zambia, Lusaka, Zambia; 6 Africa Centre of Excellence for Infectious Diseases of Humans and Animals, School of Veterinary Medicine, University of Zambia, Lusaka, Zambia; 7 Department of Disease Control, School Veterinary Medicine, University of Zambia, Lusaka, Zambia

**Keywords:** bovine brucellosis (contagious abortion), dairy cattle herds, seroprevalence, knowledge, attitudes and practices, Malawi

## Abstract

There is paucity of *Brucella* prevalence data in Malawi. For this reason, a cross-sectional study was conducted, from 06 January 2020 to 27 February 2020, to estimate the seroprevalence of brucellosis in dairy cattle herds amongst smallholder farmers, government and private dairy farms in the southern region. A total of 529 serum samples were screened for anti-*Brucella* antibodies using the Rose Bengal test (RBT) and a competitive enzyme-linked immunosorbent assay (cELISA). A pre-tested electronic (Epicollect tool, Wellcome Sanger Institute, United Kingdom) questionnaire was administered to 378 smallholder farmers to assess their knowledge, attitudes and practices towards brucellosis. Descriptive statistics were used to analyse the data in Microsoft Excel^®^ and Statistical Package for Social Sciences (SPSS^®^) version 21. No animal tested positive for presence of anti-*Brucella* antibodies, indicating 0% prevalence (individual and herd levels). The majority (94.2%; 95% confidence interval [CI]: 91.8–96.5) of smallholder farmers had never heard about brucellosis. Furthermore, assisting during parturition without protective equipment (41.3%; 95% CI: 36.3–46.2) and using bulls for breeding (75%; 95% CI: 70.2–78.9) were amongst the common risk practices that were identified. We could not detect brucellosis in this study that indicates the disease could be very rare or even absent in the dairy cattle herds of the southern region of Malawi. However, further *Brucella* studies need to be conducted in cattle, small livestock, wildlife and humans to document the true status of brucellosis in the country. Brucellosis surveillance, monitoring, awareness and preventive measures are required to maintain this favourable situation.

## Introduction

Brucellosis is an infectious bacterial disease that is caused by *Brucella* spp. and affects animals, with humans being accidental hosts (Corbel 2006). It is an emerging zoonotic disease that poses a threat to both livestock production and public health (Chota et al. 2016). In livestock, brucellosis results in reduced productivity, abortions and weak offspring and can be a major setback to both national and international livestock trade (Chota et al. 2016). The disease is usually asymptomatic in young animals and non-pregnant females (OIE 2018). In cattle and small ruminants, pregnant adult females develop placentitis usually resulting in late-term abortions following infection with *Brucella melitensis* or *Brucella abortus*. Signs of brucellosis in dairy cows are abortion, retention of placenta, swollen joints and bursae. Abortion is often the only sign observed. Not all infected cows abort although they may nevertheless spread the infective agent (Du Preez & Du Preez 2018; OIE 2018). Brucellosis in humans is characterised by intermittent fever, generalised pain (WHO/FAO/OIE 2004) and an influenza-like syndrome that is often misdiagnosed or under reported in Africa (Asakura et al. 2018). These may be slight or severe and the disease may be acute or chronic (of protracted duration). The incubation period (from infection to first symptoms) extends over 5–30 days. A very wide range of symptoms, many of which are common to other diseases, are displayed by persons with brucellosis. The disease may affect a person over several years. Medical consultation and early treatment are recommended (Du Preez & Du Preez 2018). It is debilitating and requires prolonged treatment, with a combination of antibiotics (Kunda et al. 2010).

Brucellosis has a worldwide distribution, but it is well controlled in most high income countries (Ducrotoy et al. 2015; Hadush & Pal 2013). The epidemiology of human brucellosis has drastically changed over the past decade because of sanitary, socioeconomic and political reasons, together with the evolution of international travel (Pappas et al. 2006). The geographical distribution of brucellosis is constantly changing, with new foci emerging or re-emerging (Kiros, Asgedom & Reta 2016). The epidemiology of brucellosis in livestock, particularly in sub-Saharan Africa, is not well understood and the information is often missing, inadequate or biased (Kiro et al. 2016). According to Musallam et al. (2019), *Brucella* spp. circulates amongst dairy cattle supplying milk to urban consumers in West and Central Africa, posing a serious public health concern (Table 1).

**TABLE 1:** Brucellosis prevalences in peri-urban dairy herds in Western and Central African countries.

Countries	Location	Prevalences (%)	95% (CI)
Togo	Lome	62.0	55.0–69.0
Mali	Bamako	32.5	28.0–37.0
Burundi	Bujumbura	14.7	9.4–20.8
Cameroon	Bameda	12.6	7.6–21.9
Burkina Faso	Ouagadougou	3.0	1.0–9.1
Cameroon	Ngaoundere	2.3	1.0–7.0
Senegal	Thies	1.3	0.1–5.3
Niger	Niemey	1.2	0.08–5.3
Senegal	Dakar	0.2	0.01–1.7
Senegal	Niakhar	< 0.04	0.0

*Source*: Musallam, I.I., Ndour, A.P., Yempabou, D., Ngong, C.C., Dzousse, M.F., Mouiche- Mouliom, M.M. et al., 2019, ‘Brucellosis in dairy herds: A public health concern in the milk supply chains of West and Central Africa’, *Acta Tropica* 197, 105042. https://doi.org/10.4102/j.actatropica.2019.105042

CI, confidence interval.

In Uganda, Makita et al. (2011) reported the adjusted herd (6.5%) and individual (5.0%) seroprevalences in urban and peri-urban areas of Kampala, whilst others reported seroprevalences of 1.2% (Nguna et al. 2019) and 21.5% (Kashiwazaki et al. 2012). In Kenya, a recent study by Kairu-Wanyoike et al. (2019) found a seroprevalence of 3.47% in Garissa and Tana River counties in northeast Kenya, whilst a similar study in Democratic Republic of the Congo (DRC) found an individual seroprevalence of 27.3% (Patrick et al. 2018). In Rwanda, Ndaziguraye et al. (2018) found high individual seroprevalences that ranged from 0.0% to 28.6% in different regions.

There are limited accurate data available on the prevalence of bovine brucellosis in southern Africa, as most reports are based on non-representative laboratory results. In South Africa, the absence of brucellosis in cattle, goats and dogs in the Mnisi community, bordering wildlife reserves with multiple wildlife species infected with brucellosis in the east of the country, has been recently documented in a setting with vaccination, fencing and movement control (Simpson et al. 2017); although a recent study in cattle slaughtered at the Gauteng province abattoirs, in the centre of South Africa, reported a 5.5% seroprevalence (Kolo et al. 2019). In the Nabibe province of Angola, the seroprevalence of brucellosis in animals and herds was found to be 14.96% and 40.10%, respectively (Mufinda, Boinas & Nunes 2017) whilst in Zambia, the individual prevalences of 7.9%, 14.3% and 18.7% (Chimana et al. 2014), 8.7% and 19% (Muma et al. 2006) were reported. A recent study in selected districts of Zambia by Mfune et al. (2021) reported individual and herd prevalences in ranges of 0.0% – 7.3% and 0.0% – 21.1%, respectively. In Zimbabwe, seroprevalences of 9.9% and 5.6% were found (Gomo et al. 2012; Matope et al. 2011), whilst Mozambique reported a 9.7% seroprevalence in wildlife-livestock interface areas (Tanner et al. 2014). In Namibia, an overall prevalence of 0.01% and the true prevalence of 0% were found in dairy farms between 2011 and 2014 (Madzingira & Sezuni 2017).

In Malawi, brucellosis is considered to be non-endemic and no human cases have been documented (Pappas et al. 2006). In 1986, a study by Bedard found a seroprevalence of 0.3% in cattle in Malawi (Bedard, Martin & Chinombo 1993), whilst a recent study by Tebug et al. (2014) in the Northern region of Malawi found a seroprevalence of 7.7% in dairy cattle. Importantly, the brucellosis prevalence is not well known in the southern region of Malawi, especially the Blantyre Agricultural Development Division (BLADD), which has the largest dairy herd population in the country. There is no wildlife-livestock interaction in the study area (Mulanje, Blantyre, Thyolo and Chiradzulo: BLADD) and moreover, the animals are on a cut-and-carry management system. Furthermore, although dairy farmers appear to be knowledgeable about tuberculosis as a zoonotic disease, little is known about brucellosis and farmers still practice high-risk behaviours (Tebug et al. 2014). It is against this background of inadequate research and surveillance data that this study was conducted in the southern region of Malawi to estimate the seroprevalence of brucellosis in the dairy herds and assess the associated knowledge, attitudes and practices amongst the farmers.

## Materials and methods

### Study area

**FIGURE 1:**
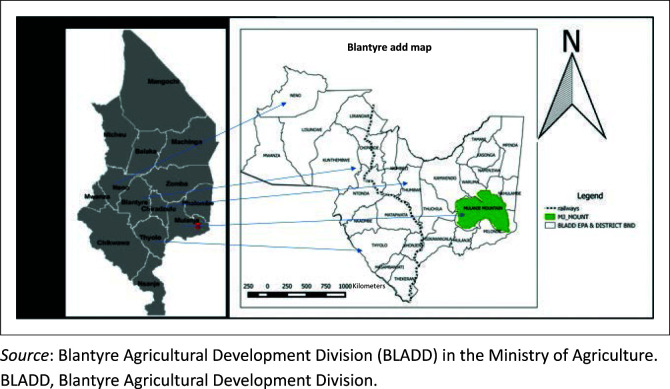
Map of the study area (Blantyre Agricultural Development Division in the southern region of Malawi).

The study was conducted in the southern region of Malawi in BLADD. The area shares borders with Mozambique and comprises seven districts, namely Mulanje (15.9974° S, 35.32466° E), Thyolo (16.0457° S, 35.28806° E), Chiradzulo (15.8196° S, 35.21621° E), Blantyre (15.8642° S, 35.0488° E), Mwanza, Neno (15.3533° S, 34.79627° E) and Phalombe (Figure 1). Five out of the seven districts were selected randomly using an Emergency Nutrition Assessment (ENA) for SMART 2011 (Erhardt et al. 2015). Two districts with a small population size of less than 0.5% were excluded. The selected five districts had 99.5% of the total animal population in the study area. The first four districts (Mulanje, Thyolo, Chiradzulo and Blantyre), constitute the BLADD milk producing area, which had 70.0% of the total dairy herds and the highest milk production in Malawi. Many dairy animals were kept by smallholder farmers using zero grazing with cut-and-carry management systems of feeding. Private and government dairy farms practiced cut-and-carry and paddock grazing, respectively. The total population of the dairy herds in this study area was 73 272 dairy animals, out of which 10 461 and 62 781 were pure breeds and crossbreeds, respectively (Apes 2018/2019). Holstein, Friesians, Jersey and Malawi zebu crosses from these pure breeds are kept in this area.

### Sample size determination and sampling strategy

All milk bulking groups (MBGs) were identified as target populations and households were selected randomly to determine the number of farmers in each MBG from each district. All dairy cattle herds were included in this study. Farmers who were inaccessible (less than 10%) because of geographical terrain, flooding and rainy season were replaced by the ones who could be reached within the MBGs. Milk bulking groups or farms were taken as herds, which consisted of homogenous basic sample units. Additional samples were purposively collected from private farms, bulls from the Shire Highland Milk Producers’ Association (SHIMPA) farm and active breeding bulls from the nearest households. The assumptions in the sample calculation were sensitivity (0.9), specificity (0.99), desired precision (0.05), significance level (95%) and assumed prevalence was 7.7% (Tebug et al. 2014). The EpiTools epidemiological calculators (http://epitools.ausvet.com.au/) was used to calculate the sample size. The estimated sample size was 131 households (farmers). However, to increase the power (hence increased sample size) and considering the structure of the herds, ENA for SMART 2011, developed by Juergen Erhardt in collaboration with Michael Golden, John Seaman and Oleg Bilukha, in 2015 (www.nutrisurvey.net/ena/ena.html) was used to calculate the sample size for households, animals and proportions of households for each district. The following assumptions were incorporated in ENA for SMART: assumed prevalence of 7.7%, population size (73 277 dairy cattle), desired precision (3% as recommended by the developer), design effect (1.5 with low heterogeneity), the herd size (three animals per farmer), percentage of cows or animals above 2 years (42%) and non-response households (3%). This generated a sample size of 495 animals and 450 households. A total of 529 animals were sampled and 378 farmers were interviewed. The inclusion criteria were dairy cattle that were more than 24 months old and heifers were not tested. Brucellosis vaccinations had never been conducted in the study area.

### Sample and data collection

Blood samples were collected from the animals’ jugular or coccygeal vein into plain pre-labelled vacutainer tubes. The tubes were stored in cooler boxes with ice packs and transported to the Blantyre Regional Veterinary Laboratory where the serum was separated by centrifuging at 4500 revolutions per minute (rpm) for 5 min. Sera was stored in 2 mL serum tubes at −20 °C until laboratory analysis.

A pre-tested electronic questionnaire (Epicollect tool, Wellcome Sanger Institute, United Kingdom) was administered to the farmers to collect information on their knowledge, attitudes and practices concerning brucellosis. The collected information included knowledge on brucellosis and zoonoses, mode of brucellosis transmission, milk consumption practices, age of the respondent, level of education, sex, breeding and management systems. After completion of the interview, the Excel^®^ sheet was generated, which was then exported to SPSS^®^ for analysis.

### Laboratory analysis of sera samples

Two independent serological tests were performed, namely the Rose Bengal test (RBT) and a competitive enzyme-linked immunosorbent assay (cELISA). All serum samples (529) were tested using the RBT and 88 randomly selected samples (from the 529 negative samples) on cELISA.

#### Rose Bengal test

All 529 serum samples were screened using the RBT manufactured by IDvet innovative Diagnostics^®^, Grabels, France according to the manufacturer’s instructions. The procedure and interpretations were performed as described in the OIE Terrestrial Manual (OIE 2018).

#### Competitive enzyme-linked immunosorbent assays

Serum samples (*n* = 88) were randomly selected and subjected to the cELISA test using (SVANOVIR^®, Uppsala, Sweden^) *Brucella*–Ab cELISA, (Boerhringer Ingelheim Svanova, Sweden) kit. This was carried out and interpreted according to the manufacturer’s instructions. Details of the procedure are described by Sagamiko et al. (2018).

### Data analysis

Data were entered into a Excel spreadsheet (Microsoft Excel^®^ 2010 version, Redmond, United States) and exported to Statistical Package for Social Sciences (SPSS^®^) version 21(International Business Machines Corporation [IBM], United States), where descriptive statistics were generated for categorical parameters. Descriptive statistics, that is, frequencies and proportions were computed and presented using tables.

### Ethical considerations

Ethical clearance was sought from and granted by the Animal Health Committee under the Department of Animal Health and Livestock Development (DAHLD) in the Ministry of Agriculture and Food Security (reference number: DAHLD/AHC/01/2019). The farmers or owners gave verbal informed consent before the interview and sample collection. Participants were assured of the confidentiality and anonymity regarding the given information.

## Results

### Seroprevalence of bovine brucellosis

The overall herd and individual animal seroprevalence for brucellosis in the study area was 0%. All 529 and 88 samples originating from 378 herds, MBGs and farms tested negative on the RBT test and cELISA, respectively.

### Knowledge, attitudes and practices of farmers towards brucellosis and other zoonotic diseases

Out of the 378 dairy farmers interviewed, 94.2% (95% confidence interval [CI]: 91.8–96.5) did not know or had never heard about brucellosis (Table 2). Only 51.6% (95% CI: 46.2–56.3) had heard and knew about dairy-related zoonotic diseases such as tuberculosis (51.3%) and brucellosis (5.8%; 95% CI: 3.4–8.1). Half of the respondents (50.8%; 95% CI: 45.7–55.8) did not know that brucellosis or other zoonotic diseases could be transmitted from dairy animals to humans, whilst 118 respondents (31.2%; 95% CI: 26.5–35.8) knew that consumption of contaminated raw milk was a mode of transmission for zoonotic diseases. Meat (13.0%; 95% CI: 9.6–16.3) was ranked second from milk as a possible mode of transmission of zoonotic diseases amongst the responses. Contact (1.6%; 95% CI: 0.3–2.8) and discharges (3.4%; 95% CI: 1.5–5.2) were the least mentioned possible modes of transmission. Most of the respondents consumed boiled milk (99.2%; 95% CI: 98.3–100.0) followed by unboiled (0.5%; 95% CI: 0.2–1.2) and soured milk (0.3%; 95% CI: 0.0–1.6).

**TABLE 2:** Potential risk factors and practices of brucellosis in study area.

Variable (potential factor)	Level	Sample percentage (%)	95% CI
Famer’s level of education	Primary	68.8	64.1–73.4
	Secondary	22.0	17.8–26.1
	Tertiary	3.0	1.2–4.7
	No formal Education	6.3	3.8–8.7
Cut-and-carry	Yes	99.2	98.3–100.0
	No	0.8	0.0–1.6
Communal grazing management	Yes	0.8	0.0–1.6
	No	99.2	98.3–100.0
History of abortion	Yes	16.9	13.1–20.6
	No	83.1	79.3–86.8
Artificial insemination	Yes	25.4	21.0–29.7
	No	74.6	70.2–78.9
Assisting parturition without protective attire	Yes	41.3	36.3–46.2
	No	58.7	53.7–63.6
Importation of replacement stock from other countries	Yes	0.8	0.0–1.6
	No	99.2	98.3–100.0
Consumption of unboiled and soured milk	Yes	0.8	0.0–1.6
	No	99.2	98.3–100.0
Brucellosis knowledge	Yes	5.8	3.4–8.1
	No	94.2	91.8–96.5

*n* = 378.

CI, confidence interval.

### Identification of potential risk factors

There was no associated risk factor identified in the study because the prevalence was 0%. However, some potential (known) risk factors were identified and many had a moderate to high percent (Table 2).

### Demographic data of participants

More than 70.0% of the smallholder farmers had either no formal education (6.3%; 95% CI: 3.8–8.7) or attended primary school (68.8%; 95% CI: 64.1–73.4). Less than 25% of the farmers had academic qualifications higher than secondary school education (22%; 95% CI: 17.8–26.1) and certificate (1.9%) or degree above (1.1%). There were more female participants (66.4%; 95% CI: 61.6–71.1) in the study. The average herd size was three animals per household.

## Discussion

### Seroprevalence of bovine brucellosis

The seroprevalence of brucellosis in our study area was 0.0%. It is worth noting that our results are contrary to findings by Tebug et al. (2014), who reported a seroprevalence of 7.7%, in dairy cattle herds in the northern region of Malawi. In northern Malawi, cattle are majorly sourced from Zambia and South Africa, countries which have reported bovine brucellosis (Tebug et al. 2014). Our study is consistent with findings by Bedard et al. (1993), who reported a 0.3% seroprevalence in Malawi, yet no positive cases in dairy cattle herds in the southern region of Malawi, where minimal contact amongst the crossbred dairy animals was reported. Although brucellosis could not be excluded, control programmes utilising vaccination were found to be unnecessary (Bedard et al. 1993). The unchanging results between the Bedard et al. (the study was conducted in 1986), and our study suggests that there has been no sustainable circulation of *B. abortus* between 1986 and 2020 in the region. It is important to report such results to document a favourable epidemiological situation, allowing the relevant authority of Malawi to make informed decisions regarding disease control policy in livestock. Moreover, these results are also important from the public health perspective, given that human brucellosis cases are almost always linked to the presence of *Brucella* spp. in livestock. Similarly, a study in the Mnisi community, Limpopo, South Africa, documented the absence of brucellosis in cattle, goats and dogs (Simpson et al. 2017). A notable difference between this study and ours is that vaccination was widely used in South Africa. Another study in KwaZulu-Natal, South Africa, found a varied brucellosis prevalence of between 0% and 15% (Hesterberg et al. 2008). This shows that it is possible to have absence of brucellosis in some areas and not in others within the same country. Another study in the Nyagatare District, Rwanda, reported a prevalence of more than 15%, whilst 0% was observed in Kamara in Rwanda in the same study by Ndazigaruye et al. (2018). Importantly, these studies highlight the patchy distribution of brucellosis in livestock, where the epidemiological situation may be dramatically different in neighbouring regions (Godfroid et al. 2013; Godfroid, Nielsen & Saegerman 2010). For example, in South Africa despite the favourable situation documented in the Mnisi community (borders the Kruger National Park) (Simpson et al. 2017), brucellosis in livestock (Kolo et al. 2019) and human brucellosis (Wojno et al. 2016) have been recently reported. However, it should be observed that our study was designed to estimate the seroprevalence of brucellosis, which is a preliminary step. To further document the absence of brucellosis (to confirm these findings) in the southern region of Malawi, herds have to be declared individually brucellosis-free and surveillance, including regular testing, needs to be implemented. Such strategy will rely on the collaboration of farmers, the quality of the veterinary services and the availability of diagnostic capacity.

In our study the herd size was small (the mean herd size was three animals per owner or household). Large herd sizes (Terefe et al. 2017) in many studies were observed to be a significant risk factor for *Brucella* infections (Lindahl et al. 2014). Thus, small and confined herds in the region could be amongst the epidemiological factors accounting for the observed 0% seropositivity in addition to safe replacement stock sources from low-risk areas, which were illustrated to be protective factors in other studies (Cárdenas et al. 2019). The main source of breeding stock to smallholder farmers in this study was within the MBGs and ADD (> 97.1%). The findings have shown that amongst all the smallholder farmers interviewed, no farmer imported a dairy animal directly from the *Brucella* high-risk areas or countries except the projects from NGOs in which the recommended protocol of *Brucella* screening was carried out before the importation of the breeding stock. The recommended protocol consisted of *Brucella* screening and quarantine of candidate animals intended for purchase. The herd management structure and source of replacement stock have a high impact on the occurrence of brucellosis because the source of breeding stock or the replacement of animals from farms not certified as brucellosis-free is one of the significant risk factors in the *Brucella* animal transmission cycle (Cárdenas et al. 2019).

### Knowledge, attitudes and practices of farmers towards brucellosis and other zoonotic diseases

Education, training and information are the key to ensure that animal owners and people who have contact with animals, do not get infected with zoonotic diseases (Du Preez & Du Preez 2018). The study found that there was poor knowledge on brucellosis amongst farmers. This is in line with previous findings in northern Malawi by Tebug et al. (2014) and other parts of Africa and Asia (Ran et al. 2019). In Tanzania, low knowledge, perception and practices towards brucellosis prevention were reported (Ntirandekura et al. 2018). Another study carried out in Nile Delta, Egypt (Abd El-Wahab et al. 2019) showed that more than two-thirds (67.4%) of the participants had not heard about brucellosis and in Rwanda 57.5% were unaware of brucellosis (Ndazigaruye et al. 2018). In contrast to our findings, some studies on brucellosis knowledge reported a 100.0% awareness in Jordan (Musallam, Abo-Shehada & Guitian 2015) whilst 99.3% of the respondents had heard about brucellosis in Uganda (Kansiime et al. 2015). A high percent of knowledge level (79.0%) in Kenya (Obonyo & Gufu 2015) and 60.0% in South Africa (Cloete et al. 2019).

In this study, 41.0% (95% CI: 36.3–46.2) of respondents assisted in parturition of livestock without protective attire. A study performed by Musallam et al. (2015) in Jordan, showed that assisting in animal parturition was one of the risk practices (62.0%) associated with brucellosis. Obonyo and Gufu (2015) in Kenya indicated that 76.0% of participants assisted an animal during the calving. The reported frequencies in both studies were higher than that reported in this study and were above 60.0%. Contrary to these findings, another study by Cloete in South Africa (Cloete et al. 2019) showed a lower percentage (21.7%) than these results, on household members who assisted in parturition. This lower percentage in our study might be because of the role played by farmer artificial inseminators who took a leading role in assisting parturition instead of farmers themselves. In the BLADD region, trained technicians in artificial insemination are available and assist farmers with pregnancy diagnosis and sometimes the management of dystocia. These are trained by different organisations such as Shire Highlands Milk Producers’ Association (SHIMPA), World Vision international and Department of Animal Health and Livestock Development (DAHLD).

The study has found that most of the farmers consumed boiled milk. This is contrary to a study conducted in Kenya which recorded 96% raw milk consumption in a year (Obonyo & Gufu 2015). In another study conducted in Nile Delta, Egypt, drinking raw fresh milk was an uncommon practice owing to the awareness of associated hazards (Abd El-Wahab et al. 2019). In this present study, the social-cultural practice was the main reason for low or no raw milk consumption. Furthermore, the majority of smallholder farmers in the study area used a bull or natural service for breeding, a finding that is similar to that of Ndazigaruye et al. (2018), who observed that most of the farmers used natural service. In the presence of brucellosis seropositivity, sharing of bulls between herds is one of the most risky practices (Ndazigaruye et al. 2018). The high cost, lack of access and need for repeat inseminations were the main reasons reported by the farmers for preference of use of natural service to artifical insemination.

Despite the absence of brucellosis in the dairy herds in southern Malawi, sustained surveillance is required to maintain this favourable situation. This brucellosis status can dramatically change if *B. abortus* infects dairy herds in the southern region of Malawi in the future. Indeed, the dairy cattle herds in the southern region of Malawi could be immunologically naive towards *Brucella* infection. Should *B. abortus* infect such a naive population, acute brucellosis would be seen in pregnant animals (heifers and cows, regardless of the previous number of calvings). Animal health and production and human health would be compromised and the livelihood of communities would be dramatically affected.

### Study limitations

The study was limited to dairy herds in the southern region, especially BLADD. For this reason, the results from the study cannot be generalised to the northern and central regions of Malawi and beef cattle herds in the same study area because of differences in management and geographical risk factors. Sampling milk from the MBGs would also be the better approach in estimating the *Brucella* herd prevalence in the region and this was not performed because of some resource limitations.

## Conclusion

We could not detect anti-*Brucella* antibodies in cattle in the study area and this could indicate very low prevalence levels or an absences of brucellosis in the dairy cattle herds of the southern region of Malawi. There was poor knowledge on this zoonotic disease and other zoonoses and high-risk practices were identified in the region. In the near future, brucellosis tests would have to be performed on the dairy cattle of the southern region of Malawi again to confirm whether the region is truly free of brucellosis. It is recommended that surveillance and monitoring of brucellosis is carried out consistently. In addition, increasing public health awareness and supporting measures to prevent the introduction of brucellosis in dairy cattle herds needs to be strengthened. Furthermore, serological, bacteriological and molecular studies should be carried out nation-wide (in beef and dairy cattle, wildlife, small livestock and humans) to understand the epidemiology of brucellosis in Malawi.

## References

[CIT0001] Abd El-Wahab, E.W., Hegazy, Y., El-Tras, W.F., Mikeal, A., Kapaby, A.F., Abdelfatah, M. et al., 2019, ‘Knowledge, attitudes and practices (KAPs) and risk factors of brucellosis at the human-animal interface in the Nile Delta, Egypt’, *bioRxiv*. 10.1101/607655 31309735

[CIT0002] Apes, 2018/2019, *Agricultural production estimates*, Ministry of Agriculture and Food security, DAHLD, Lilongwe.

[CIT0003] Asakura, S., Makingi, G., Kazwala, R. & Makita, K., 2018, ‘Brucellosis risk in urban and agro-pastoral areas in Tanzania’, *EcoHealth* 15, 41–51. 10.1007/s10393-017-1308-z 29344824

[CIT0004] Bedard, B.G., Martin, S.W. & Chinombo, D., 1993, ‘A prevalence study of bovine tuberculosis and brucellosis in Malawi’, *Preventive Veterinary Medicine* 16(3), 193–205. 10.1016/0167-5877(93)90066-3

[CIT0005] Cárdenas, L., Peña, L., Melo, C. & Casal, J., 2019, ‘Risk factors for new bovine brucellosisinfections in Colombian herds’, *BMC Veterinary Research* 15, 81. 10.1186/s12917-019-1825-9 30845954 PMC6404332

[CIT0006] Chimana, H.M., Muma, J.B., Samui, K.L., Hangombe, B.M., Munyeme, M., Matope, G. et al., 2014, ‘A comparative study of the seroprevalence of brucellosis in commercial and small-scale mixed dairy-beef cattle enterprises of Lusaka province and Chibombo district, Zambia’, *Tropical Animal Health and Production*, 42, 1541–1545. 10.1007/s11250-010-9604-4 20517646

[CIT0007] Chota, A.C., Magwisha, H.B., Stella, B., Bunuma, E.K., Shirima, G.S., Mugambi, G.M. et al., 2016, ‘Prevalence of brucellosis in livestock and incidences in humans in East Africa’, *African Crop Science Journal* 24(S1), 45–55. 10.4314/acsj.v24i1.5S

[CIT0008] Cloete, A., Gerstenberg, C., Mayet, N. & Tempia, S., 2019, ‘Brucellosis knowledge, attitudes and practices of a South African communal cattle keeper group’, *Onderstepoort Journal of Veterinary Research* 86(1), a1671. 10.4102/ojvr.v86i1.1671 PMC6407466 30843408

[CIT0009] Corbel, M.J., 2006, ‘Brucellosis in humans and animals’, *American Journal of Public Health and the Nations Health* 30(3), 299–300. 10.2105/AJPH.30.3.299

[CIT0010] Ducrotoy, M., Bertu, W.J., Matope, G., Cadmus, S., Conde-Álvarez, R., Gusi, A.M. et al., 2015, ‘Brucellosis in sub-Saharan Africa: Current challenges for management, diagnosis and control’, *Acta Tropica* 165, 179–193. 10.1016/j.actatropica.2015.10.023 26551794

[CIT0011] Du Preez, J.H. & Du Preez, G.P., 2018, *Animal diseases and man – Zoonoses*, Printed and bound by CTP, Cape Town, viewed 22 February 2021, from www.zoonoses.co.za.

[CIT0012] Erhardt, J., Golden, M., Seaman, J. & Bilukhu, O., 2015, *ENA for SMART*, www.smartmethodology.org., viewed 25 August 2020, from www.nutrisurvey.net/ena/ena.html.

[CIT0013] Godfroid, J., Garin-Bastuji, B., Saegerman, C. & Blasco, J.M., 2013, ‘Brucellosis in terrestrial wildlife’, *Revue Scientifique et Technique* 32(1), 27–42. 10.20506/rst.32.1.2180 23837363

[CIT0014] Godfroid, J., Nielsen, K. & Saegerman, C., 2010, ‘Diagnosis of brucellosis in livestock and wildlife’, *Croatian Medical Journal* 51(4), 296–305. 10.3325/cmj.2010.51.296 20718082 PMC2931434

[CIT0015] Gomo, C., De Garine-Wichatitsky, M., Caron, A. & Pfukenyi, D.M., 2012, ‘Survey of brucellosis at the wildlife-livestock interface on the Zimbabwean side of the Great Limpopo transfrontier conservation area’, *Tropical Animal Health Production* 44, 77–85. 10.1007/s11250-011-9890-5 21643664

[CIT0016] Hadush, A. & Pal, M., 2013, ‘Brucellosis – An infectious re-emerging bacterial zoonosis of global importance’, *International Journal of Livestock Research* 3(1), 28–34. 10.5455/ijlr.20130305064802

[CIT0017] Hesterberg, U.W., Bagnall, R., Perrett, K., Bosch, B., Horner, R. & Gummowa, B., 2008, ‘A serological prevalence study of important infectious diseases of cattle in rural areas of Kwa Zulu Natal, South Africa’, *Journal of South Africa veterinary Association* 79(1), 15–18. 10.4102/jsava.v79i1.234 18678186

[CIT0018] Kairu-Wanyoike, S., Nyamwaya, D., Wainaina, M., Ontiri, E., Bukachi, S., Njeru, I. et al., 2019, ‘Positive association between *Brucella* spp. seroprevalences in livestock and humans from a cross-sectional study in Garissa and Tana River Counties, Kenya’, *PLoS Neglected Tropical Diseases* 13(10), e0007506. 10.1371/journal.pntd.0007506 31622339 PMC6818805

[CIT0019] Kansiime, C., Mugisha, A., Makumbi, F., Mugisha, S., Rwego, I.B., Sempa, J. et al., 2015, ‘Knowledge and perceptions of brucellosis in the pastoral communities adjacent to Lake Mburo National Park, Uganda’, *BMC Public Health* 14, 242. 10.1186/1471-2458-14-242 PMC3975325 24612845

[CIT0020] Kashiwazaki, Y., Ecewu, E., Imaligat, J.O., Mawejje, R., Kirunda, M., Kato, M. et al., 2012, ‘Epidemiology of bovine brucellosis by a combination of Rose Bengal test and indirect ELISA in the five districts of Uganda’, *The Japanese Society of Veterinary Science* 74(11), 1417–1422. 10.1292/jvms.12-0164 22785123

[CIT0021] Kiros, A., Asgedom, H. & Reta, D.A., 2016, ‘A review on bovine brucellosis: Epidemiology, diagnosis and control options’, *ARC Journal of Animal and Veterinary Sciences (AJAVS)* 2(3), 8–21. 10.20431/2455-2518.0203002

[CIT0022] Kolo, F.B., Adesiyun, A.A., Fasina, F.O., Katsande, C.T., Dogonyar, B.B., Potts, A. et al., 2019, ‘Seroprevalence and characterization of *brucella* species in cattle slaughtered at Gauteng abattoirs, South Africa’, *Veterinary Medicine and Science* 5(4), 545–555. 10.1002/vms3.190 31414558 PMC6868451

[CIT0023] Kunda, J., Fitzpatrick, J., French, N., Kazwala, R., Kambarage, D., Mfinanga, G.S. et al., 2010, ‘Quantifying risk factors for human brucellosis for rural northern Tanzania’, *PLoS One* 5(4), e9968. 10.1371/journal.pone.0009968 20376363 PMC2848606

[CIT0024] Lindahl, E., Sattorov, N., Boqvist, S., Sattori, I. & Magnusson, U., 2014, ‘Seropositivity and risk factors for brucella in dairy cowsin urban and peri-urban small-scale farming in Tajikistan’, *Tropical Animal Health and Production* 46, 563–569. 10.1007/s11250-013-0534-9 24414248 PMC3936117

[CIT0025] Madzingira, O. & Sezuni, P.M., 2017, ‘Serological prevalence and public health significance of brucellosis on a dairy farm in Namibia from 2011 to 2014’, *BMC Research Notes* 10, 620. 10.1186/s13104-017-2933-x 29178929 PMC5702218

[CIT0026] Makita, K., Fèvre, E.M., Waiswa, C., Eisler, M.C., Thrusfield, M. &Welburn, S.C., 2011, ‘Herd prevalence of bovine brucellosis and analysis of risk factors in cattle in urban and peri-urban areas of the Kampala economic zone, Uganda’, *BMC Veterinary Research* 7, 60. 10.1186/1746-6148-7-60 22004574 PMC3212899

[CIT0027] Matope, G., Evison, B., Muma, J.B., Oloya, J., Madekurozwa, R.L., Lund, A. et al., 2011, ‘Seroprevalence of brucellosis and its associated risk factors in cattle from smallholder dairy farms in Zimbabwe’, *Tropical Animal Health and Production* 43, 975–982. 10.1007/s11250-011-9794-4 21327714

[CIT0028] Mfune, R.L., Mubanga, M., Silwamba, I., Sagamika, F., Mudenda, S., Daka, V. et al., 2021, ‘Seroprevalence of bovine brucellosis in selected districts of Zambia’, *International Journal of Environmental Research and Public Health* 18(4), 1436. 10.3390/ijrph18041436 33546514 PMC7913639

[CIT0029] Mufinda, F.C., Boinas, F. & Nunes, C., 2017, ‘Prevalence and factors associated with human brucellosis in livestock professionals’, *Rev Saude Publica* 51, 57. 10.1590/S1518-8787.2017051006051 28658364 PMC5493365

[CIT0030] Muma, J.B., Samui, K.L., Siamudaala, V.M., Oloya, J., Matope, G., Omer, M.K. et al., 2006, ‘Prevalence of antibodies to *Brucella* spp. and individual risk factors of infection in traditional cattle, goats and sheep reared in livestock-wildlife interface areas of Zambia’, *Tropical Animal Health and Production* 38, 195–206. 10.1007/s11250-006-4320-9 16986767

[CIT0031] Musallam, I.I., Abo-Shehada, M.N. & Guitian, J., 2015, ‘Knowledge, attitudes, and practices associated with brucellosis in livestock owners in Jordan’, *The American Society of Tropical Medicine and Hygiene* 93(6), 1148–1155. 10.4269/ajtmh.15-0294 PMC4674226 26438029

[CIT0032] Musallam, I.I., Ndour, A.P., Yempabou, D., Ngong, C.C., Dzousse, M.F., Mouiche-Mouliom, M.M. et al., 2019, ‘Brucellosis in dairy herds: A public health concern in the milk supply chains of West and Central Africa’, *Acta Tropica* 197, 105042. 10.1016/j.actatropica.2019.105042 31152725 PMC6710496

[CIT0033] Ndazigaruye, G., Mushonga, B., Kandiwa, E., Samkange, A. & Segwagwe, B.E., 2018, ‘Prevalence and risk factors for brucellosis seropositivity in cattle in Nyagatare district, Eastern province, Rwanda’, *Journal of the South African Veterinary Association* 89, a1625. 10.4102/jsava.v89i0.1625 PMC6295791 30551701

[CIT0034] Nguna, J., Dione, M., Apamaku, M., Majalija, S., Mugizi, D.R., Odoch, T. et al., 2019, ‘Seroprevalence of brucellosis and risk factors associated with its seropositivity in cattle, goats and humans in Iganga district, Uganda’, *Pan African Medical Journal* 33, a99. 10.11604/pamj.2019.33.99.16960 PMC6711673 31489077

[CIT0035] Ntirandekura, J-B., Matemba, L.E., Ngowi, H.A., Kimera, S.I. & Karimuribo, E.D., 2018, ‘Knowledge, perceptions and practices regarding brucellosis in pastoral communities of Kagera region in Tanzania’, *Journal of Advanced Veterinary and Animal Research* 5(3), 243–253. 10.5455/javar.2018.e285

[CIT0036] Obonyo, M. & Gufu, W.B., 2015, ‘Knowledge, attitude and practices towards brucellosis among Pastoral community in Kenya, 2013’, *International Journal of Innovative Research and Development* 4(10), viewed 11 May 2020, from www.ijird.com.

[CIT0037] OIE, 2018, ‘Brucellosis (*Brucella abortus, Brucella melitensis, Brucella suis*)’, in *Infection with B. abortus, B. melitensis and B. suis*’, 2018 edn., pp. 355–398, OIE Terrestrial Manual, viewed 25 November 2019, from https://www.oie.int/fileadmin/Home/eng/Health_standards/tahm/3.01.04_BRUCELLOSIS.pdf.

[CIT0038] Pappas, G., Papadimitriou, P., Akritidis, N., Christou, L. & Tsianos, E.V., 2006, ‘The new global map of human brucellosis’, *Lancet Infectious Disease* 6(2), 91–99. 10.1016/S1473-3099(06)70382-6 16439329

[CIT0039] Patrick, B.N., Ahadi, B.B., Gedéon, B., Shukuru, W.D., Gedéon, B.N. & Gitao, G.C., 2018, ‘Seroprevalence survey of brucellosis among cattle in selected districts of South Kivu province, Eastern of Dr Congo’, *Journal of Bio Innovation* 7(3), 424–434.

[CIT0040] Ran, X., Cheng, J., Wang, M., Chen, X., Wang, H., Ge, Y. et al., 2019, ‘Brucellosis seroprevalence in dairy cattle in China during 2008–2018: A systematic review and meta-analysis’, *Acta Tropica* 189, 117–123. 10.1016/j.actatropica.2018.10.002 30308207

[CIT0041] Sagamiko, F.D., Muma, J.B., Karimuribo, E.D., Mwanza, A.M., Sindato, C. & Hang’ombe, B.M., 2018, ‘Sero-prevalence of bovine brucellosis and associated risk factors in Mbeya region, Southern highlands of Tanzania’, *Acta Tropica* 178, 169–175. 10.1016/j.actatropica.2017.11.022 29191516

[CIT0042] Simpson, G., Marcotty, T., Rouille, E., Matekwe, N., Letesson, J.J. & Godfroid, J., 2017, ‘Documenting the absence of brucellosis in cattle, goats and dogs in a One Health^ interface in the Mnisi community, Limpopo, South Africa’, *Tropical Animal Health and Production* 50, 903–906. 10.1007/s11250-017-1495-1 29274056

[CIT0043] Tanner, M., Inlameia, O., Michel, A., Maxlhuza, G., Pondja, A., Fafetine, J. et al., 2014, ‘Bovine tuberculosis and brucellosis in cattle and African buffalo in the Limpopo National Park, Mozambique’, *Transboundary and Emerging Diseases* 62(6), 632–638. 10.1111/tbed.12210 24479882

[CIT0044] Tebug, S.F., Njunga, G.R., Chagunda, M.G.G., Mapemba, J.P., Awah-Ndukum, J. & Wiedemann, S., 2014, ‘Risk, knowledge and preventive measures of smallholder dairy farmers in northern Malawi with regard to zoonotic brucellosis and bovine tuberculosis’, *Onderstepoort Journal of Veterinary Research* 81(1), a594. 10.4102/ojvr.v81i1.594 24832647

[CIT0045] Terefe, Y., Girma, S., Mekonnen, N. &Asrade, B., 2017, ‘Bruc ellosis and associated risk factors in dairy cattle of Eastern Ethiopia’, *Tropical Animal Health and Production* 49, 599–606. 10.1007/s11250-017-1242-7 28176187

[CIT0046] WHO/FAO/OIE, 2004, *Report of the WHO/FAO/OIE joint consultation on emerging zoonotic diseases*, WHO/FAO/OIE, Geneva.

[CIT0047] Wojno, J.M., Moodley, C., Pienaar, J., Beylis, N., Jacobsz, L. & Nicol, M.P., 2016, ‘Human brucellosis in South Africa: Public health and diagnostic pitfalls’, *South African Medical Journal* 106(9), 883–885. 10.7196/SAMJ.2016.v106i9.11020 27601111

